# Post-traumatic diaphragmatic ruptures – 12 years experience

**DOI:** 10.1186/1749-8090-8-S1-O76

**Published:** 2013-09-11

**Authors:** C Tunea, O Burlacu, V Voiculescu, G Cozma, I Miron, A Nicola, A Nicodin

**Affiliations:** 1 Department of Thoracic Surgery, Emergency Municipal Hospital Timisoara, Romania; 2 University of Medicine, Timisoara, Romania

## Background

Undetected post-traumatic trans-diaphragmatic hernias due to once minor diaphragmatic lacerations can lead over time to serious complications. Early recognition and treatment are mandatory to minimize complication.

## Methods

We reviewed retrospectively the charts of 18 patients admitted between 2001and 2012, presenting with diaphragmatic lacerations post trauma. We noted the traumatic mechanism, involvement of other organs, diagnostic methods, surgical approach, and postoperative stay. All patients were submitted to open surgery. The surgical repair of the diaphragm was performed using double layer in separate stitches. We used average with standard deviation and correlation Pearson index.

## Results

We had a 0,2 ratio female/male, mean age 41.88±17,08 years. The mechanism of injury was 5 stabbing wounds, 5 rib fractures, 4 crash syndrome, and 4 decelerations. 15 cases were operated on as an immediate emergency, 3 cases were delayed. The surgery was: thoracic in 15 and thoraco-abdominal in 3 cases. We noted 3 complications. Pearson index for age-postoperative days was 0.241.

## Conclusions

Due to the serious short and long term complications and lesion associations, post-traumatic diaphragmatic lacerations must be approached early and by a multidisciplinary surgical team. In a poli-traumatized patient with severe bleeding often minimally invasive approach is in our opinion not sufficient.

